# The Subgingival Plaque Microbiome, Systemic Antibodies against Bacteria and Citrullinated Proteins following Periodontal Therapy

**DOI:** 10.3390/pathogens10020193

**Published:** 2021-02-10

**Authors:** Emily Davison, William Johnston, Krystyna Piela, Bob T. Rosier, Michael Paterson, Alex Mira, Shauna Culshaw

**Affiliations:** 1 Oral Sciences, College of Medical, Veterinary and Life Sciences, Dental School, University of Glasgow, Glasgow G12 8QQ, UK; emilysarahdavison@gmail.com (E.D.); w.johnston.1@research.gla.ac.uk (W.J.); km.piela@gmail.com (K.P.); Michael.paterson@glasgow.ac.uk (M.P.); 2 Division of Dentistry, Medical University of Lodz, 92-213 Lodz, Poland; 3 The Foundation for the Promotion of Health and Biomedical Research (FISABIO), 46020 Valencia, Spain; rosier_bob@gva.es (B.T.R.); mira_ale@gva.es (A.M.)

**Keywords:** periodontitis, periodontal therapy, *Porphyromonas gingivalis*, *Aggregatibacter actinomycetemcomitans*, anti-citrullinated protein antibodies, subgingival plaque, inflammation

## Abstract

Periodontitis (PD) shows an association with rheumatoid arthritis (RA) and systemic inflammation. Periodontal pathogens, namely *Porphyromonas gingivalis* and *Aggregatibacter actinomycetemcomitans*, are proposed to be capable of inducing citrullination of peptides in the gingiva, inducing the formation of anti-citrullinated protein antibodies (ACPAs) within susceptible hosts. Here, we sought to investigate whether periodontal treatment influenced systemic inflammation and antibody titres to *P. gingivalis*, *A. actinomycetemcomitans*, *Prevotella intermedia* and ACPA in 42 systemically health patients with periodontal disease. Subgingival plaque and serum samples were collected from study participants before (baseline) and 90 days after treatment to analyse the abundance of specific bacteria and evaluate anti-bacterial antibodies, C-reactive protein (CRP), tumour necrosis factor α (TNF-α), interleukin 6 (IL-6) and ACPA in serum. Following treatment, all patients showed reduced periodontal inflammation. Despite observing a weak positive correlation between CRP and IL-6 with periodontal inflammation at baseline, we observed no significant reductions in any indicators of systemic inflammation 90 days after treatment. In contrast, anti-*P. gingivalis* IgG significantly reduced post-treatment (*p* < 0.001, Wilcoxon signed rank test), although no changes were observed for other antibody titres. Patients who had detectable *P. gingivalis* in subgingival plaques had significantly higher anti-*P. gingivalis* IgG and ACPA titres, suggesting a potential association between *P. gingivalis* colonisation and systemic antibody titres.

## 1. Introduction

Periodontitis (PD) is a chronic inflammatory disease characterised by diverse dysbiotic microbial communities and an aberrant immune response, resulting in irreversible destruction of the tooth supporting apparatus and ultimately tooth loss [1]. Although previously thought to be limited to the oral cavity, PD has been linked to systemic diseases with complex pathogenesis involving inflammation and immune dysregulation, such as diabetes, cardiovascular disease and rheumatoid arthritis (RA) [2,3,4]. However, the biological mechanisms underlying these links remain incompletely understood.

In PD, there is a marked shift in the composition of the subgingival plaque microbiome compared with the microbial community found in periodontal health. This shift likely reflects the inflammatory milieu that changes the environmental conditions, and the presence of key periodontitis-associated bacteria that directly affect the behaviour of neighboring organisms [5]. Such species include *Porphyromonas gingivalis* and *Aggregatibacter actinomycetemcomitans*. These key pathogens cluster to form discrete bacterial complexes that can remodel the healthy microbial population into dysbiosis [6].

RA is a debilitating autoimmune disease resulting in joint destruction, with systemic effects that ultimately results in a 54% higher observed mortality rate compared to that of the general population [7]. Both RA and PD share genetic and environmental risk factors, as well as exhibiting similar pathophysiological processes. Both diseases involve cellular infiltration at an inflammatory focus, express similar cytokine profiles, involve activation of matrix metalloproteinase and result in irreversible destruction of bone and connective tissue [8]. In RA, cytokines including interleukin-6 (IL-6) and tumour necrosis factor alpha (TNF-α) play a pivotal role in driving systemic inflammation and acute phase proteins, such as C-reactive protein (CRP), become increased and can be used diagnostically [9]. Autoantibodies known as anti-citrullinated peptide antibodies (ACPAs) target peptides in the joints, which have been modified to contain citrulline [10]. ACPAs are an important predictive factor for the development of RA, as well as the disease progression and prognosis of the disease [11]. ACPAs can be detected several years prior to the clinical onset of RA, suggesting that the joints may not be the triggering site for autoantibody generation [12,13,14]. ACPAs have been detected in PD patients and studies have suggested an association between PD and citrullination of host and bacterial peptides in the gingiva [15,16]. *P. gingivalis* and *A. actinomycetemcomitans* have been identified as possible bacterial triggers for the formation of ACPAs as both bacteria have been shown to expose the immune system to citrullinated epitopes. It has been hypothesised that in genetically predisposed patients, exposure to citrullination in the gingival tissues could induce a breach of immunological tolerance, resulting in the development of autoantibodies [16,17,18].

The presence of serum anti-bacterial IgG antibodies directed against periodontal pathogens has been suggested as a potential serological diagnostic tool for PD [19] Several studies have shown elevated levels of these antibodies in patients with PD compared with healthy controls, especially directed against *P. gingivalis* [20,21,22]. In contrast, others suggest anti-bacterial antibodies directed against periodontal pathogens are only modestly associated with PD [20]. Furthermore, the true sensitivity and specificity of the assays employed to detect such antibodies remains an area of controversy, and it is currently unclear whether these antibodies indicate periodontal disease status, current exposure, previous exposure or possibly cross-reaction with other bacterial epitopes, and thus their diagnostic use remains limited [19,20,21,22].

Non-surgical periodontal therapy (NST) involves the mechanical debridement of supra- and subgingival plaque and aims to disrupt microbial biofilms and reduce inflammation. NST has been shown to be an effective method of improving clinical periodontal parameters [23], and there is a noted increase in presence of health-associated bacteria following treatment [24,25]. The complex relationships between the oral microbiome, local inflammation in the mouth, systemic inflammatory and antibody responses remain poorly understood. Understanding how these factors interact could help understand the link between periodontitis and systemic diseases and potentially identify novel preventative or therapeutic opportunities. We sought to investigate whether—alongside changing clinical and microbiological changes following periodontal treatment —there are changes in systemic inflammation, anti-bacterial antibody titres, and anti-citrullinated protein antibody titres. The aim of this longitudinal study was to investigate the effect of routine NST on systemic anti-bacterial titre, markers of systemic inflammation, ACPA titre and the subgingival plaque microbiome, and explore any associations between these parameters in a cohort of patients with PD. Patients with PD only, rather than PD and RA, were chosen to avoid the confounding factors associated with RA-related treatments.

## 2. Results

### 2.1. Study Cohort and Clinical Improvement

Thirty females and 12 males received NST with varying numbers of treatment visits (ranging from one to six). Across all patients, NST resulted in significant improvement in all periodontal parameters. This included reductions in periodontal pocket depth (median (Q1, Q3): 3.59 mm (3.08–4.16) to 2.70 mm (2.56–3.02), *p* < 0.001 Wilcoxon Signed Rank test) and percentage of pockets ≥5 mm (27.50% (18.75–41.75) to 9.00% (5.75–12.25), *p* < 0.001 Wilcoxon Signed Rank test), with a treatment response commensurate with a recently published systematic review [23]. Similar improvements were observed for the periodontal inflamed surface area (PISA) (1224.60 mm^2^ (754.80–1687.08) to 138.85 mm^2^ (65.52–293.88), *p* < 0.001 Wilcoxon Signed Rank test), which was subsequently used as an indicator of clinical inflammation.

### 2.2. Association between Clinical Parameters and Systemic Markers

**Figure 1:**
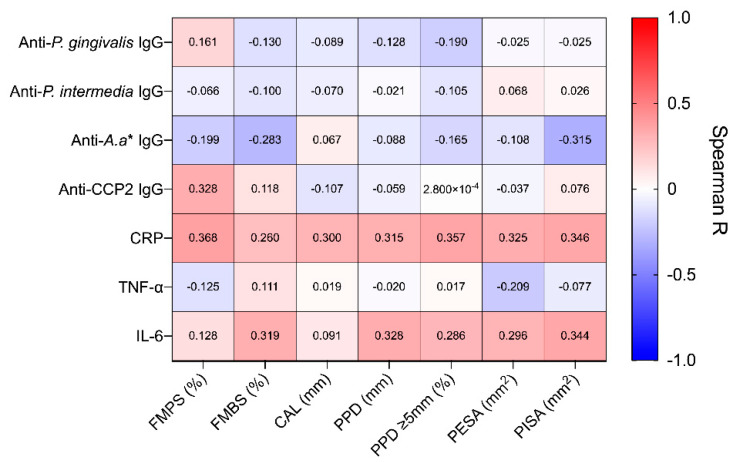
Correlation between systemic markers and periodontal clinical parameters at baseline. Correlations were performed using Spearman–Rho method, with individual R values shown above. P-values were adjusted for multiple comparisons using the False Discovery Rate (5%) method, no significant correlations were found following this adjustment. FMPS, full-mouth plaque score; FMBS, full-mouth bleeding score; CAL, clinical attachment level; PPD, periodontal probing depth, PPD ≥ 5 mm; percentage of pockets ≥ 5 mm; PESA, periodontal epithelial surface area; PISA, periodontal inflamed surface area.

Prior to treatment, we observed a large spread in the baseline disease severity of included patients. For example, the percentage of pockets ≥5 mm ranged from 7.00% to 65.00%, and PISA values ranged from 45.60 mm^2^ to 3655.90 mm^2^ across all patients. Given this heterogeneity, we investigated whether systemic inflammatory markers and antibody titres were associated with periodontitis severity (Figure 1). There were weak positive associations between CRP and several periodontal indices. This included full-mouth plaque index (r = 0.368, 95% CI: 0.063 to 0.612, *p* = 0.016), percentage of pockets ≥5 mm (r = 0.357, 95% CI: 0.050 to 0.602, *p* = 0.020) and PISA (r = 0.346, 95% CI: 0.038 to 0.594, *p* = 0.025). Similarly, a weak association was observed between IL-6 and PISA (r = 0.344, 95% CI: 0.036 to 0.593, *p* = 0.025). Interestingly, a weak negative correlation was observed between anti-*A. actinomycetemcomitans* IgG and PISA (r = −0.315, 95% CI: −0.574 to 0.001, *p* = 0.045), while a weak positive correlation was observed between ACPA and full-mouth plaque score (FMPS) (r = 0.328, 95% CI: 0.017 to 0.581, *p* = 0.034). However, following correction and adjustment for multiple comparisons, no association remained statistically significant (all *p* (adjusted) > 0.05). Notably, there were no significant associations between IgG antibodies directed against *P. gingivalis* and clinical indices.

### 2.3. Longitudinal Alterations in Systemic Inflammatory Markers

**Figure 2:**
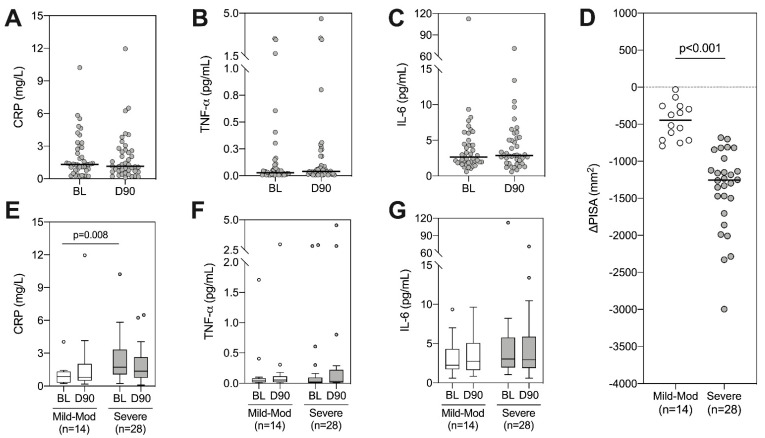
Longitudinal analysis of systemic inflammatory markers following periodontal therapy. (**A**) CRP, (**B**) TNF-α and (**C**) IL-6 were quantified at baseline (BL) and day 90 (D90). Wilcoxon signed rank tests were performed, all *p* > 0.05. (**D**) Patients were split into mild-moderate or severe based on their baseline PISA value [26] and the change in PISA following treatment is displayed, *p* < 0.001 using Mann–Whitney test. Longitudinal analysis of CRP (**E**), TNF-α (**F**) and IL-6 (**G**) was analysed within each group using Wilcoxon signed rank tests (all *p* > 0.05), between-group comparisons refer to Mann–Whitney tests (raw *p*-values shown when *p* < 0.05).

Having observed a weak relationship between periodontal and systemic inflammation, we next sought to assess whether NST impacted systemic inflammatory markers (CRP, IL-6, TNF-α). However, despite consistent reductions in periodontal inflammation, we observed no significant change in any systemic marker following treatment (Figure 2A–C). Given that patients within this study displayed varying degrees of disease severity at baseline, and as such, the reduction in periodontal inflammation varied following treatment, we hypothesized that these results may be skewed by patients with only a small change in their periodontal inflammation. To evaluate whether this impacted alterations in systemic inflammatory markers, we split patients into mild/moderate and severe disease based on PISA [26]. Patients with severe disease at baseline showed significantly greater reduction in PISA after treatment, indicating a larger reduction in local inflammatory load (Figure 2D). Consistent with previous results, those with severe disease had significantly higher levels of CRP at baseline (Figure 2E, *p* = 0.008 Mann–Whitney test). However, to our surprise, there were no significant changes in CRP following periodontal treatment in either the severe disease group or in the mild/moderate disease group (Figure 2E–G).

### 2.4. Subgingival Plaque Colonisation by P. gingivalis, A. actinomycetemcomitans and P. intermedia

**Figure 3:**
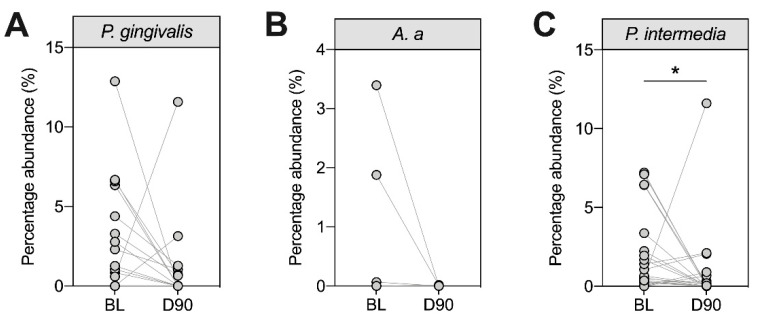
Longitudinal assessment of selected periodontal pathogens in subgingival plaque samples at baseline (BL) and day 90 (D90). Subgingival plaque samples from all patients were analysed at baseline and day 90 following non-surgical periodontal therapy (NST) for the proportion of *P. gingivalis* (**A**), *A. a* (**B**) and *P. intermedia* (**C**) using 16S rRNA sequencing (*n* = 42). Each dot shows one patient. Patients who were negative at BL and D90 were not included. Statistics refer to Wilcoxon signed rank tests where * *p* < 0.05. A. a: *Aggregatibacter actinomycetemcomitans.*

We next assessed the relative abundance of three periodontal pathogens (*P. gingivalis*, *A. actinomycetemcomitans* and *P. intermedia*) in the subgingival plaque using 16S rRNA sequencing (Figure 3). Although all patients in this study had periodontitis, our data suggested varying colonization patterns by *P. gingivalis*, *A. actinomycetemcomitans* and *P. intermedia*. At baseline, 13 patients had detectable levels of *P. gingivalis* (31%), four had detectable levels of *A. actinomycetemcomitans* (9.5%) and 24 had detectable levels of *P. intermedia* (57%). In general, the proportions of these bacteria decreased 90 days after treatment, although this reduction was only statistically significant for *P. intermedia* (Figure 3C).

### 2.5. Longitudinal Alterations in Antibody Titres

**Figure 4:**
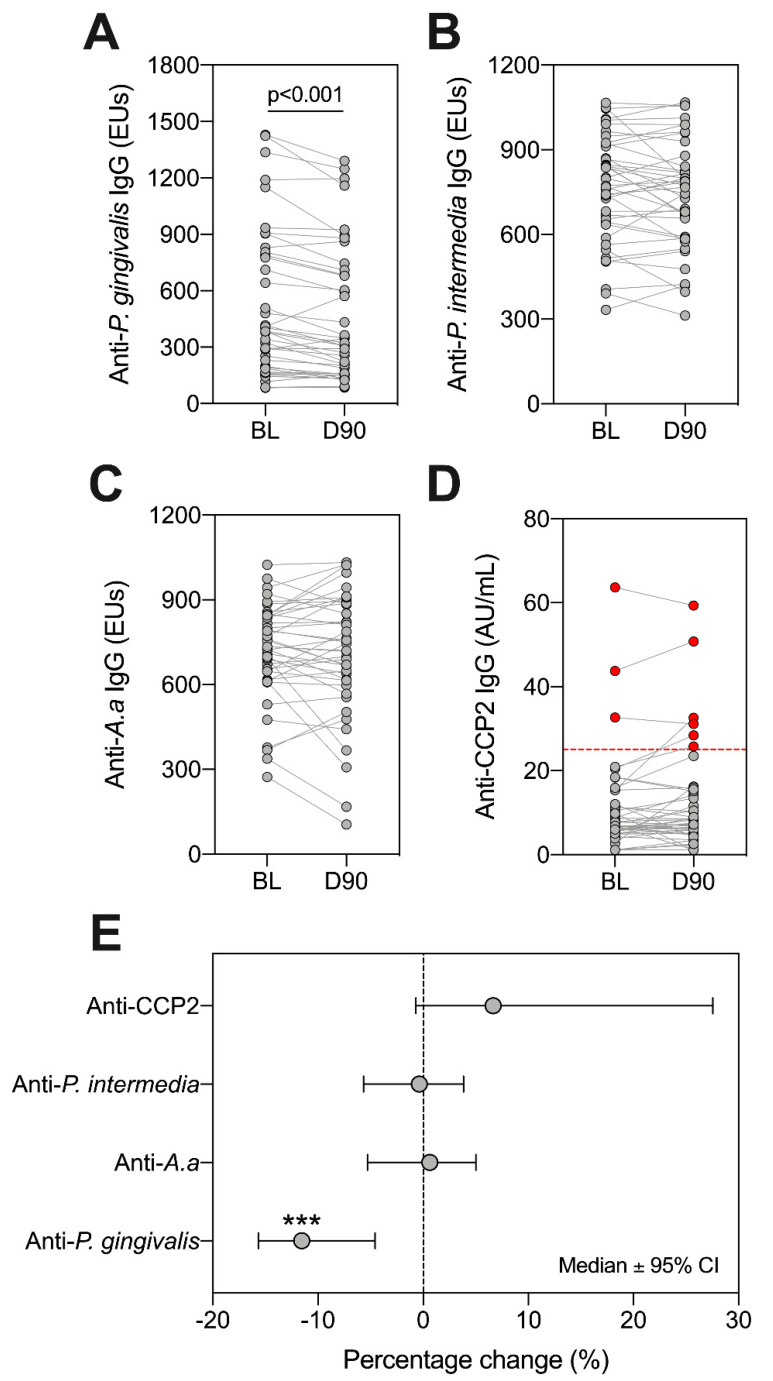
Assessing changes in serum IgG against ACPA and periodontal bacteria at baseline (BL) and day 90 (D90). Antibodies in serum reactive against *P. gingivalis* (**A**), *P. intermedia* (**B**), A.a (**C**) and CCP2 (**D**) were investigated. ‘ACPA-positive’ patients are highlighted as red dots, with the positivity threshold (25 AU/mL) indicated by dotted red line (**D**). Lines between points indicate the same patient. Statistics are Wilcoxon signed rank test, where *** *p* < 0.001. The percentage change across all patients is highlighted (**E**), where dots indicate medians and error bars are 95% confidence intervals. A and D: *n* = 42, B and C: *n* = 41. A. a: *Aggregatibacter actinomycetemcomitans*.

Having identified varying colonization patterns of *P. gingivalis*, *A. actinomycetemcomitans* and *P. intermedia*, we next sought to establish whether antibody titres directed against these organisms, and ACPAs, changed following treatment. We observed a significant reduction in anti-*P. gingivalis* IgG at day 90 (Figure 4A, *p* < 0.001 Wilcoxon Signed Rank test). In contrast, there were no significant changes in antibody titres for anti-*P. intermedia*, anti-*A. actinomycetemcomitans* (Figure 4B,C), *p* > 0.05 for all, Wilcoxon Signed Rank test). Using the ACPA assay diagnostic positivity threshold (25 AU/mL), we found that three patients were ‘ACPA-positive’ at baseline (7.14%), and six patients were positive at day 90 (14.29%) (Figure 4D). Only anti-*P. gingivalis* antibodies showed a reduction following treatment (Figure 4E).

### 2.6. Anti-Bacterial Antibodies and ACPAs according to Periodontal Disease Severity

**Figure 5:**
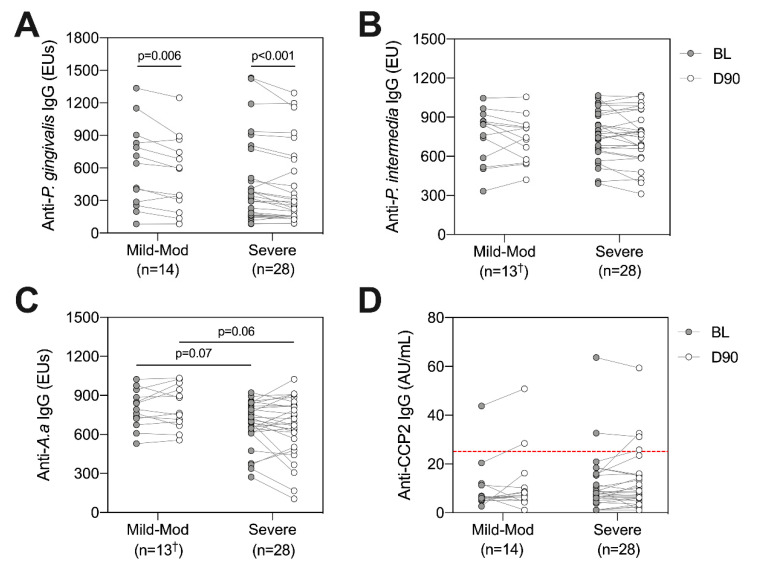
Investigating anti-bacterial antibodies and ACPAs at baseline (BL, grey circles) and day 90 (D90, white circles) according to PISA groups. Patients were split into mild-moderate (*n* = 14^†^) or severe (*n* = 28) based on their baseline PISA value [26]. Anti-bacterial antibodies directed against *P. gingivalis* (**A**), *P. intermedia* (**B**), *A. a* (**C**) and ACPAs (**D**) were compared between groups at each timepoint, and longitudinally within groups following NST. The ACPA positivity threshold (25 AU/mL) is indicated by dotted red line (D). Lines connect individual patients at each timepoint, between group comparisons refer to Mann–Whitney test, within group comparisons refer to Wilcoxon signed rank test. Raw p-values are displayed. A.a: *Aggregatibacter actinomycetemcomitans*. ^†^ One patient had limited serum volume and was not included in analysis of anti-*P. intermedia* or anti-*A.a* IgG, *n* = 13.

Similar to analysis of systemic inflammatory markers, we evaluated whether baseline disease severity and the degree of inflammation reduction following treatment impacted anti-bacterial antibodies and ACPAs. Using the PISA grouping method described previously, levels of antibodies directed against *P. gingivalis*, *P. intermedia*, *A. actinomycetemcomitans* and ACPAs were compared between ‘mild-moderate’ and ‘severe’ groups at baseline and day 90, and longitudinally within each group following treatment (Figure 5). Matching with analysis of the entire cohort, reductions in anti-*P. gingivalis* IgG were observed following treatment in both the mild-moderate (*p* = 0.006, Wilcoxon signed rank test) and severe groups (*p* < 0.001, Figure 5A). In contrast, no significant difference in anti-*P. gingivalis* IgG was observed between groups at baseline (*p* = 0.35, Mann–Whitney test) or day 90 (*p* = 0.41, Figure 5A). Interestingly, no longitudinal alterations were observed within groups for anti-*P. intermedia*, anti-*A. actinomycetemcomitans* or ACPAs, suggesting that a larger reduction in periodontal inflammation did not impact changes in antibody titres (Figure 5B–D). In line with the weak negative correlation between PISA and anti-*A. actinomycetemcomitans* IgG, a trend was observed whereby the severe PISA group showed lower anti-*A. actinomycetemcomitans* IgG, albeit this did not reach statistical significance at either timepoint (*p* = 0.07 at baseline, *p* = 0.06 at day 90, Mann–Whitney test, Figure 5C).

### 2.7. P. gingivalis Colonisation and Antibody Titres

**Figure 6:**
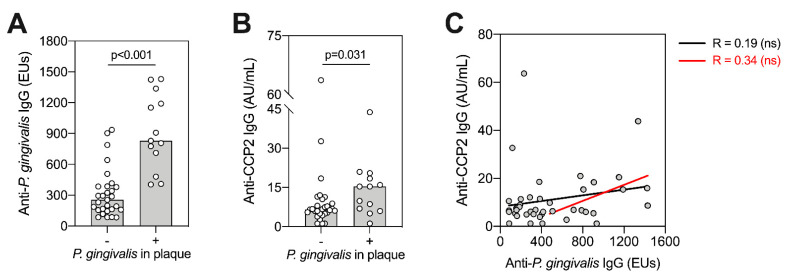
Association between ACPA and *P. gingivalis*. Patients were split according to whether *P. gingivalis* was detectable (+) or undetectable (-) using 16S rRNA sequencing (**A**,**B**). Bars display medians with individual values (*n* = 42). Comparisons between groups were performed using Mann–Whitney U-tests, with individual p-values shown. (**C**) Anti-*P. gingivalis* IgG antibodies were correlated against anti-CCP2 IgG antibodies using Spearman–Rho. This was performed across all patients (black line, *n* = 42), or including only seropositive patients (red line, *n* = 16), ns means no significant difference observed (*p* > 0.05). Seropositivity was determined as antibody titres at least two standard deviations above the mean antibody titre from an independent periodontally healthy control group, set at 435.07 ELISA units (EUs).

Given differences in *P. gingivalis* colonization, we compared ACPA and anti-*P. gingivalis* IgG titre with subgingival *P. gingivalis* plaque status (positive or negative) and assessed whether levels of serum anti-*P. gingivalis* IgG titre were associated with ACPA titre at baseline (Figure 6). We found that patients positive for *P. gingivalis* in subgingival plaques had significantly higher anti-*P. gingivalis* (Figure 6A, *p* = <0.001, Mann–Whitney test), and ACPA titres (Figure 6B, *p* = 0.031, Mann-Whitney test). However, this was not observed in patients with *A. actinomycetemcomitans* and *P. intermedia* positivity in subgingival plaque (data not shown). When correlated among all patients, we observed no direct association between anti-*P. gingivalis* IgG and ACPA (Figure 6C, black lines). When assessing only *P. gingivalis* “seropositive” patients, this trend appeared stronger but remained non-significant (Figure 6C, red lines).

### 2.8. ACPA Association with Subgingival Plaque Microbiome

**Figure 7:**
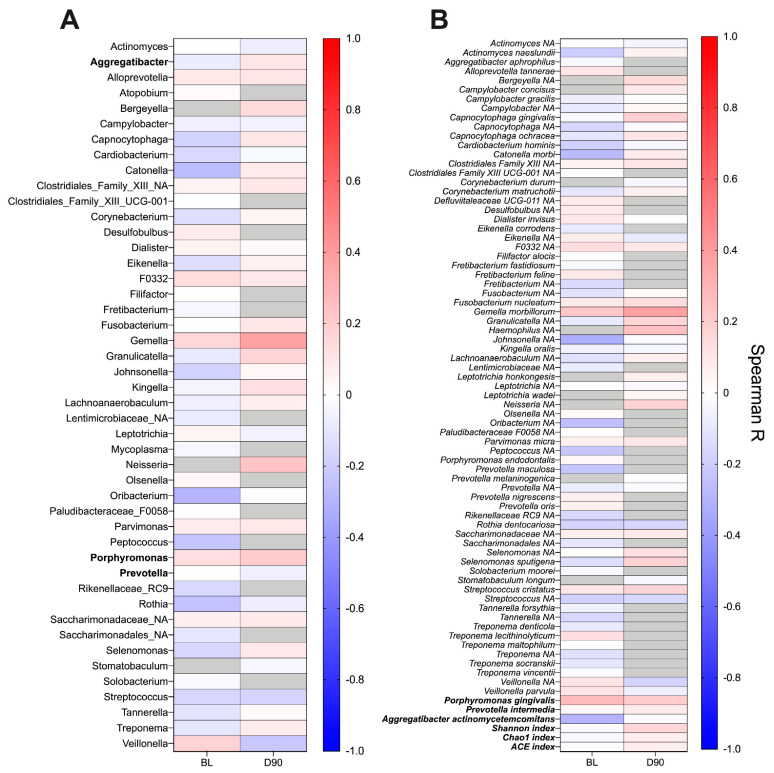
Heatmap presenting correlations between the relative abundance of genera (**A**) and species (**B**) in the subgingival plaque and serum ACPA titre. Correlation analysis was performed at baseline (BL) and day 90 (D90) using Spearman–Rho. Genera and species were included if they were present in ≥50% of samples at each timepoint. Grey boxes indicate organisms not meeting this abundance threshold and were thus not included in the correlation analysis. In addition, post-hoc correlation analysis was performed using species of interest in this study and alpha-diversity indexes (bold). No significant correlations were observed at either timepoint.

Finally, taking a non-bias approach, we investigated whether ACPA titres correlated with the relative abundance of any bacterial genera or species in the subgingival plaque at baseline or day 90 (Figure 7A,B). Assessing correlations among abundant species (see methods section), no positive or negative correlation was observed with serum ACPA titre (Figure 7B). A similar finding was obtained at genus-level (Figure 7A). In addition, no association was found between the abundance of species of interest in this study (*P. gingivalis*, *P. intermedia* and *A. actinomycetemcomitans*) or with the taxonomic diversity of the microbiome at baseline or day 90.

## 3. Discussion

This study uniquely investigated the relationship of the ACPA serum response with anti-bacterial serum antibodies and the subgingival plaque microbiome in a periodontitis cohort before and after NST. Our data suggest that *P. gingivalis* carriage may be associated with anti-*P. gingivalis* IgG antibodies and ACPAs; findings that support previous studies of RA patients and their first-degree relatives (FDR-RA) cohorts, and supporting previously proposed links between *P. gingivalis* and ACPA [27,28,29,30,31].

Limited studies exist investigating ACPA in periodontitis patients following NST, (two to the authors’ knowledge), and none have evaluated the entire subgingival plaque microbiota [27,32]. In our cohort, three patients were ACPA positive at baseline, and six patients were ACPA positive at day 90, representing 7.14% and 14.29% of patients respectively. This value is higher than the general population, where it is believed that ACPA positivity is roughly 2.8% using the same CCP2 assay [33]. However, in our cohort, the anti-CCP2 titre in ACPA-positive individuals is considerably lower compared with the average titre in RA-positive patients. It is recognised that the higher the ACPA titre, the more likely the individual is to be RA-positive or develop RA. The meaning of our low-titre ACPA-positive individuals therefore remains uncertain, and further follow up is needed to ascertain whether these ACPA-positive individuals may eventually go on to develop RA and how their ACPA titre varies over time. Longer term effect of NST on ACPA titre has never been documented. Previous studies have assessed ACPA titre at baseline and 6 months or 8 weeks only [27,32]. Periodontitis is a chronic lifelong condition with many patients relapsing. Further follow up could address this gap in the literature to investigate the long-term effects of periodontal therapy and maintenance on ACPA titre.

In contrast to previous studies, we did not observe a significant decrease in ACPA titre post-NST. Both Yang et al. and Lappin et al. conducted similar studies investigating the effect of NST on ACPA in PD patients following NST [27,32]. Both found ACPA titre significantly reduced; Yang in all patients and Lappin only in non-smokers. We also did not see a significant decrease in CRP, IL-6 or TNF-α, in contrast with Yang et al. where a decrease in TNF-α was observed. The follow up period for these studies was 8 weeks and 6 months compared with our timeframe of 90 days, and this could account for some differences between study results. Interestingly, we did observe a weak association between CRP/IL-6 and PISA prior to correction for multiple comparisons. It would be useful in further studies to increase the study population to discern whether this finding is significant. Similarly, for patients with high baseline PISA scores, CRP levels did not change despite substantial reductions in periodontal inflammation. It has been previously shown that changes in CRP post-treatment are most impactful in patients with co-morbidities [34]. Thus, it is possible that since our patients were generally fit and well, we were less likely to observe changes in serum CRP.

One limitation of our study is that we did not have a control group with periodontitis that did not have treatment, as this study focused on the effect on antibody titre and microbiome before and after NST. Furthermore, ACPA positivity has been previously related to the presence and severity of periodontitis [35]. It is possible that the association seen in literature reflects *P. gingivalis* carriage, rather than the severity of periodontal disease. To account for this, future studies should consider swabbing in areas such as tongue and tonsil for *P. gingivalis* carriage.

Our data suggest that *P. gingivalis* presence in subgingival plaques may influence ACPA titre. Although whole microbiome analysis did not identify a correlation between any particular species with ACPA, there appeared to be a weak correlation between ACPA titre and anti-*P. gingivalis* IgG, albeit not statistically significant. A recent meta-analysis showed that RA patients have an increased immune response to *P*. *gingivalis* reflected through increased anti-*P. gingivalis* IgG titre compared with healthy and PD controls [36]. *P. gingivalis* expresses the enzyme peptidylarginine deiminase (PPAD), a bacterial virulence factor unique to *P. gingivalis* that citrullinates epitopes [18]. PPAD is hypothesised to create a citrullinated antigen that acts as a systemic immunogen responsible for ACPA formation in PD patients. Further investigations should identify if PPAD activity correlates with ACPA in PD patients. As different *P. gingivalis* strains have different PPAD activity, there may be variation between patients that could influence ACPA titre [27]. ACPA-positive PD patients could also be tested for ACPA specificities, e.g., CEP, citFib, and a non-citrullinated native protein control, which our study lacks. This will help further determine whether the ACPA-positive patients found in our PD cohort are specific for bacterial citrullinated epitopes, or are cross-reactive with human citrullinated peptide.

Our results indicate that the presence, rather than the relative abundance of *P. gingivalis* in subgingival plaques is associated with systemic antibody titres. Whilst the results from this current study are based on 16S rRNA analysis of a single site, the presence of *P. gingivalis* in subgingival plaque has been shown to associate with serum IgG antibodies directed against *P. gingivalis* and gingipains (RgpB) in several previous studies [37,38].

Our second key finding is that periodontal inflammation and anti-*P. gingivalis* IgG significantly decrease post-NST, and these findings are commensurate with previous results [30,39,40]. The literature on changes in anti-*P. intermedia* IgG and anti *A. actinomycetemcomitans* IgG are less consistent, and our data showed no significant change for either [19,39,41,42,43]. One possible reason for this is that anti-*A. actinomycetemcomitans* and anti-*P. intermedia* IgG titre reflects history of colonisation rather than active infection: only four patients tested positive for *A. actinomycetemcomitans* in subgingival plaques, but all patients had detectable levels of anti-*A. actinomycetemcomitans* IgG. This raises the question of the usefulness and accuracy of using serum antibodies to identify periodontal disease-associated bacteria. We observed no correlation between antibody titres and clinical disease severity, which is in contrast with previous studies [44]. There is not a single well-defined protocol for assays to detect anti-bacterial antibodies, with studies using single strains, multiple strains or bacterial virulence factors, such as PPAD, or LtxA for detection [45,46,47]. These different approaches present a challenge when comparing studies investigating ACPAs and serum antibodies. Until the clinical usefulness of anti-bacterial IgG is discerned and standardised, clinical examination remains the most accurate way to classify and identify periodontal disease.

In conclusion, the data presented show evidence for a relationship between *P. gingivalis* colonisation and ACPA titre. If ACPA positivity in periodontitis patients precedes the development of RA, this is an important clinical finding as patients could be identified to be treated earlier.

## 4. Materials and Methods

### 4.1. Study Population, Treatment and Clinical Examination

This study received ethical approval (REC reference: 14/LO/2064) and was conducted in accordance with the Declaration of Helsinki 2013. Forty-five patients were recruited for this study. This was based upon a pilot study, showing that the minimum detectable change in antibody titres pre- and post-treatment in a study population of 34 would be 0.5 SD (based on 80% power at the 5% significance level) [27]. Forty-two patients were included in analysis after excluding 3 individuals who were diagnosed with a systemic disease throughout the trial period. Patients suffering from PD, defined as probing pocket depths ≥5 mm on 2 or more teeth at non-adjacent sites, were selected for inclusion. Additional inclusion criteria included providing written informed consent and males or females aged ≥18 years, generally fit and well. Exclusion criteria included patients with known or suspected risk for arthritis, diabetes, tuberculosis, hepatitis B, HIV infections, history of bleeding diathesis, systemic antibiotics in last 3 months, requiring interpreter or non-English language written material to provide written informed consent.

The number of treatment visits varied and depended on patient preference for treatment schedule, and baseline disease severity. Periodontal parameters were assessed at baseline (BL) and 90 days following periodontal treatment (D90). Periodontal parameters were assessed at six sites per tooth, including full-mouth plaque scores (FMPS), full-mouth bleeding scores (FMPS), periodontal probing depths (PPD) and clinical attachment levels (CAL). Following measurements, the periodontal epithelial surface area (PESA) and periodontal inflamed surface area (PISA) were calculated as previously described [48] and used as indicators of periodontal inflammation. We classified 14 patients as having moderate periodontal disease and 22 having severe disease using PISA values based off a classification method described by Leira [27]. Briefly, patients with PISA scores of >934.71 mm^2^ were classed as severe, 521.56–934.71 mm^2^ as moderate and <531.56 mm^2^ as mild.

### 4.2. Sample Collection and Processing

Subgingival plaque and serum samples were collected prior to periodontal measurements at baseline and day 90 as previously described [49]. Plaque was collected from the deepest pocket in each quadrant using a curette. For serum samples, blood was collected by venepuncture into sterile collection tubes (Vacuette, Greiner bio-one, Gloucestershire, UK) and centrifuged at 2500 RPM for 10 min. All samples were stored at −80 °C until analysis.

### 4.3. CCP2 ELISA

ACPAs in serum was tested at baseline and day 90 using the commercially available CCP2 ELISA kit Immunoscan CCPlus^®^ (EuroDiagnostica AB, Malmö, Sweden) in accordance with manufacturer’s instructions. All samples were assayed in duplicate, and the average value was taken to calculate ACPA titres. The CCP2 assay has a diagnostic cut off for positivity at >25 U/mL. The limit of detection (LOD) for this assay was 2.295 AU/mL, calculated using the blank mean + SD*5. ACPA was <LOD in 7 samples (4 at baseline, 3 at day 90), which were given as LOD/2 (1.14743923) for statistical analysis.

### 4.4. Anti-Bacterial Antibody Titres

Serum IgG antibody titres against periodontal bacteria (*P. gingivalis* W83, *P. intermedia* ATCC 25611, and *A. actinomycetemcomitans* ATCC 43718) were determined as outlined previously with minor modifications [27,50]. In brief, Immulon 1B low-binding microtitre plates (Thermofisher, Loughborough, UK) were coated overnight with heat-killed organisms in carbonate bicarbonate buffer pH 9.6 (Sigma Aldrich, Gillingham, UK). Coating concentrations of each organism were determined using healthy and periodontitis control serum and were selected based on maximising signal-to-noise ratio. After coating, wells were washed and blocked using 0.1% foetal bovine serum in PBS (Gibco, Thermofisher, Loughborough, UK). Serum dilutions were then added to wells ranging from 1/50 to 1/400 for patient and control samples, with each sample being assayed in duplicate. Bound antibody was detected using biotin conjugated anti-human IgG antibodies, extravidin peroxidase and TMB substrate (Sigma Aldrich, Gillingham, UK). Antibody titres are presented as ELISA units calculated as previously described [27,50].

### 4.5. Analysis of Inflammatory Markers

Levels of serum CRP were determined by immunoturbidometry using the Cobas C311 analyser (Cobas, Roche Diagnostic, Mannheim, Germany). Serum IL-6 and TNF-α were determined using high-sensitivity ProQuantum immunoassays, performed using a StepOne plus real-time PCR analyser (Thermofisher, Loughborough, UK). Each sample was assayed in duplicate. Limit of detection (LOD) for ProQuantum assays were: IL-6 (0.119 pg/mL) and TNF-α (0.012 pg/mL). IL-6 and CRP were detected in all samples (*n* = 84). TNF-α was <LOD in 17 samples (10 at baseline, 7 at day 90). For statistical analysis, these values were given as LOD/2.

### 4.6. Bacterial 16S rRNA Sequencing

One site from each patient was selected for 16S rRNA sequencing, with subgingival plaque the same site being analysed at baseline and day 90. DNA from subgingival plaque collected from all patients at baseline and day 90 was extracted using the MagNA Pure LC DNA isolation kit (Roche Diagnostics, Mannheim, Germany), and measured with the QubitTM 3 Fluorometer (Thermofisher, Loughborough, UK), and then sequenced using an Illumina MiSeq sequencer and taxonomy assigned as reported elsewhere [49,51].

### 4.7. Data Analysis

Statistical analyses of clinical characteristics, serum inflammatory markers and anti-bacterial antibody titres were performed using GraphPad PRISM version 8.0 (La Jolla, California, CA, USA). For comparison of clinical parameters at baseline and day 90, Wilcoxon sign rank test was used for non-normally distributed data. For comparison of ACPA and anti-bacterial antibody titres, Wilcoxon Signed Rank tests were used. For correlation analysis between clinical characteristics, ACPA and anti-bacterial antibody titres, Spearman–Rho correlation coefficient was used. For analysis of ACPA and anti-bacterial antibody titres with subgingival plaque positivity and ACPA with anti-bacterial antibody positivity, Mann–Whitney U-test was used. Correlations between abundant genera and species and ACPA titres were performed in R (version 3.4) using Spearman–Rho correlation coefficient. Abundant genera and species were defined as those present in at least 50% of samples at baseline (relevant for baseline correlations) or day 90 (relevant for day 90 correlations), with an abundance greater than five times the smallest percentage above zero.

## Data Availability

Data is available upon reasonable request from the corresponding author.
